# Different types of sensory nerve endings in the urinary bladder of mice arising from dorsal root ganglia (DRG) at the thoracolumbar region of vertebral column

**DOI:** 10.1007/s00441-026-04083-4

**Published:** 2026-06-08

**Authors:** Nick J. Spencer, Melinda A. Kyloh, Lee Travis, Vladimir P. Zagorodnyuk, Luke Grundy, Tim Hibberd

**Affiliations:** Visceral Neurophysiology Laboratory, College of Medicine and Public Health, Flinders Health & Medical Research Institute, Bedford Park, SA 5042 Australia

**Keywords:** Sensory nerve endings, Dorsal root ganglia, Urinary bladder, Spinal afferent

## Abstract

The urinary bladder is innervated by two anatomically distinct populations of sensory neurons originating from dorsal root ganglia (DRG): one from the thoracolumbar (TL) and another from the lumbosacral (LS) spinal region. While we previously characterized the morphology and distribution of sensory endings arising from LS DRG, the terminals of TL afferents remain undefined. In this study, we employed anterograde neuronal tracing to map these endings in mice. Following bilateral injection of dextran biotin into T12–L3 DRG and a 7–9-day recovery, whole bladders were processed to visualize spinal afferent axons and their calcitonin gene-related peptide (CGRP) immunoreactivity. We identified four morphological types of sensory endings, similar to those described for LS DRG: simple and complex types in both the sub/urothelium and the detrusor muscle. However, their distribution was fundamentally different. Most TL afferents (79%) terminated within the sub/urothelium, with a minor proportion (21%) in the detrusor. This pattern is the inverse of LS innervation, which predominantly targets the detrusor (81%). Furthermore, nearly all TL endings (96%) were peptidergic (CGRP +). This stark anatomical and neurochemical segregation suggests that TL and LS spinal afferent populations are functionally specialized. We propose that the dense TL innervation of the sub/urothelium may constitute a protective surveillance system sensitive to chemical irritation and inflammation, whereas the predominant LS innervation of the detrusor is likely specialized for detecting mechanical stretch during bladder filling.

## Introduction

Spinal afferent neurons encode sensory signals arising from the bladder. They comprise two anatomically distinct populations originating from either lumbosacral or thoracolumbar dorsal root ganglia (DRG) (Christianson & Davis 2010; Grundy et al. 2019; Janig 1986; Janig & Morrison 1986; Zagorodnyuk et al. 2007). Our prior work in mice characterized the morphology and distribution of sensory endings arising from lumbosacral DRG at the level of L5-S1 (Spencer et al. 2018). Rodents (e.g., mice), provide a good model for understanding the spinal sensory innervation of humans, but there are some important differences between species. Mice typically have 34 pairs of spinal nerves (8 cervical, 13 thoracic, 6 lumbar, and 3 coccygeal) while humans have 31 pairs of spinal nerves (8 cervical, 12 thoracic, 5 lumbar, 5 sacral, and 1 coccygeal). Therefore, while L6 spinal nerve in mice provides a rich spinal afferent innervation to visceral organs like the bladder (Spencer et al. 2018) and distal colon (Spencer et al. 2014), there is no L6 vertebrae or spinal nerve in most human spinal cords.

Progress in the field of visceral sensation has been hindered by a lack of methods for selectively labelling spinal afferent axons and identifying their detailed nerve terminal morphology. To overcome this, we used a survival surgery technique developed previously from our laboratory that enabled direct DRG neuronal tracer injections in mice, enabling high-resolution identification and mapping of individual spinal afferent nerve endings in viscera (Kyloh & Spencer 2014; Spencer et al. 2014). Applying this technique to the bladder for the first time, we identified three distinct ending types from lumbosacral DRG which we characterized as simple, branching, and complex types (Spencer et al. 2018); all of which were found within the detrusor muscle (81% of all endings) while only simple types were found in the urothelium or sub/urothelium (19% of all endings) (Spencer et al. 2018).

The aim of this study was to identify and characterize sensory endings in the urinary bladder that arise from the second major population—thoracolumbar DRG (T12-L3) —and determine how they compare with those previously identified from lumbosacral levels (Spencer et al. 2018).

## Methods

### Surgical procedure and tissue processing

Experiments were approved by the Animal Welfare Committee of Flinders University of South Australia (ethics #784/11 & #861–13). All protocols were carried out in strict accordance with the recommendations in the Guide for the Care and Use of Laboratory Animals of the National Health & Medical Research Council (NH&MRC) of Australia. Under isoflurane anesthesia, a ~ 10 mm dorsal incision was made in young (< 60-day-old) C57BL/6 mice to expose the thoracolumbar DRG (T12-L3). Biotinylated dextran (10–20%, D1956, Molecular Probes) was injected bilaterally into single DRG from a pulled glass micropipette (TW150-4, WPI) advanced by micromanipulator (Narishige M-4001002). Injections (< 100 nL per DRG) were performed with a custom nitrogen-driven spritz system (5–10 min, 1 s pulses at 0.3 Hz, 10–15 psi). The muscle and skin were sutured closed, and animals recovered for 7–9 days. Following euthanasia by isoflurane overdose, the bladder was removed via laparotomy and pinned mucosal-side-up as a sheet preparation in PBS. Bladders were fixed overnight in 4% paraformaldehyde (PFA), then cleared with three 10-min washes in dimethyl sulfoxide (DMSO; RCL Labscan). Tissues were subsequently washed in phosphate-buffered saline (PBS) (3 × 10 min) before a 2-h incubation in Cy3-conjugated streptavidin (Jackson Immunoresearch, 016–160-084; RRID AB_2337244; 1:200 in antibody diluent). The diluent contained 10% sodium azide, NaCl, Na₂HPO₄, and NaH₂PO₄·H₂O. Finally, tissues were washed again in PBS (3 × 10 min), then mounted on a glass slide in 100% phosphate buffered glycerol (pH 8.6) for initial inspection of axonal labelling. Following confirmation of axon labelling, whole-mount bladders were washed in PBS (3 × 10 min) and blocked for 1 h (PBS containing 1% BSA, 5% normal donkey serum, and 1% Triton X-100). Tissues were incubated for 48 h in rabbit anti-CGRP primary antibody (1:2000 in PBS + 1% Triton X-100, Table 1), washed again (PBS, 3 × 10 min), and then incubated in donkey anti-rabbit Cy5 secondary antibody (Jackson ImmunoResearch Lot #105748, RRID:AB_2340607, 1:200). Finally, preparations were slide-mounted in 100% buffered glycerol.

**Table 1:** Antibody and its descriptions

Antibody	Description of immunogen	Source, host species, cat.#, clone or lot#, RRID	Conc	RRID
CGRP	Rat αCGRP, full length protein, peptide sequence: HSCATATCVT HRLAGLLSRS GGVVKNNFVPTNVGSEAF-NH2	Peninsula Laboratories/ra Rabbit/Cat.# IHC6006 /Lo Lot#040826–2, AB_2314156	1:2000	AB_2314156

### Image capture and analysis

Slides were imaged using an Olympus IX71 microscope with epifluorescence and high-specificity filter sets (Chroma Technology). Images were captured at × 4, × 10, and × 20 magnification with a CoolSNAP fx CCD camera (Photometrics) controlled by AnalySIS FIVE software (v5.0, Olympus) and saved as TIFF files. To confirm colocalization, afferent endings labelled with neuronal tracer (Cy3 channel) were first identified and imaged. The identical location was then imaged using the Cy5 filter to verify CGRP immunoreactivity. Images were analysed to classify each ending's morphology, location, and CGRP immunoreactivity, with results tabulated in Microsoft Excel. Using ImageJ software, we measured the axon width and the dimensions of its varicosities. A total of 10 random varicosities was selected and measured along each spinal afferent axon terminal and the mean measurements determined. The width of varicosities (referred to as short axis) and their length (long axis of the varicosities) were compared between afferent classes by Tukey’s multiple comparisons tests following 2-way ANOVA (main factors: tissue layer and afferent class; both *p* > 0.05, with no significant pairwise differences). Analysis was performed in Graphpad Prism software (version 11.0.0). A *p*-value < 0.05 was considered statistically significant.

#### Spinal afferent morphological classification

Spinal afferent nerve endings were classified into three morphological types based on our established scheme (Spencer et al. 2018). Simple-type endings featured single, minimally branched axons without specialized terminals. Branching-type endings comprised a single axon that arborized into multiple parallel, varicose axons, analogous to intramuscular arrays (IMAs) described in other viscera (Kyloh & Spencer 2014; Spencer et al. 2014; Zagorodnyuk et al. 2001). Complex-type endings were identified by extensive, disorganized axonal bifurcations with no preferential orientation relative to the bladder wall (Spencer et al. 2018).

## Results

**Fig. 1:**
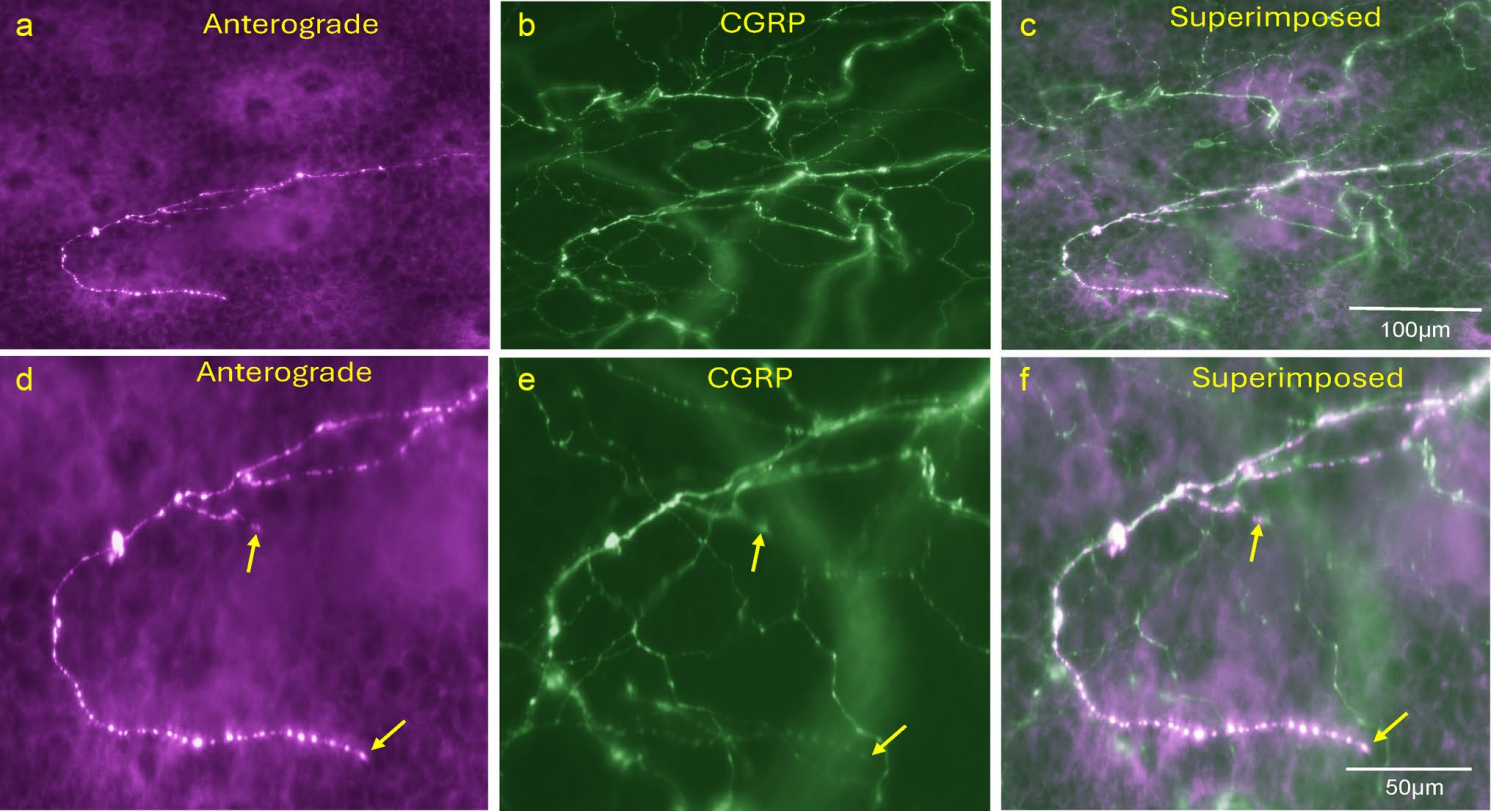
Simple type spinal afferent in the sub/urothelium.** a** A varicose axon ramifies within the sub/urothelium, with binuclear urothelial cells visible in the background. The terminal ending is unspecialized, matching the size of other axonal varicosities. **b** CGRP immunoreactivity of the field in **a**. **c** Superimposition of **a** and **b**. **d** Higher magnification of the axon in **a**. Arrows indicate varicose terminals and a branch point where a single axon bifurcates briefly before terminating. **e** CGRP immunoreactivity of the field in **d**. **f** Superimposition of **d** and **e**; arrows confirm the ending is peptidergic (CGRP +) and mark the site of axon termination

**Fig. 2:**
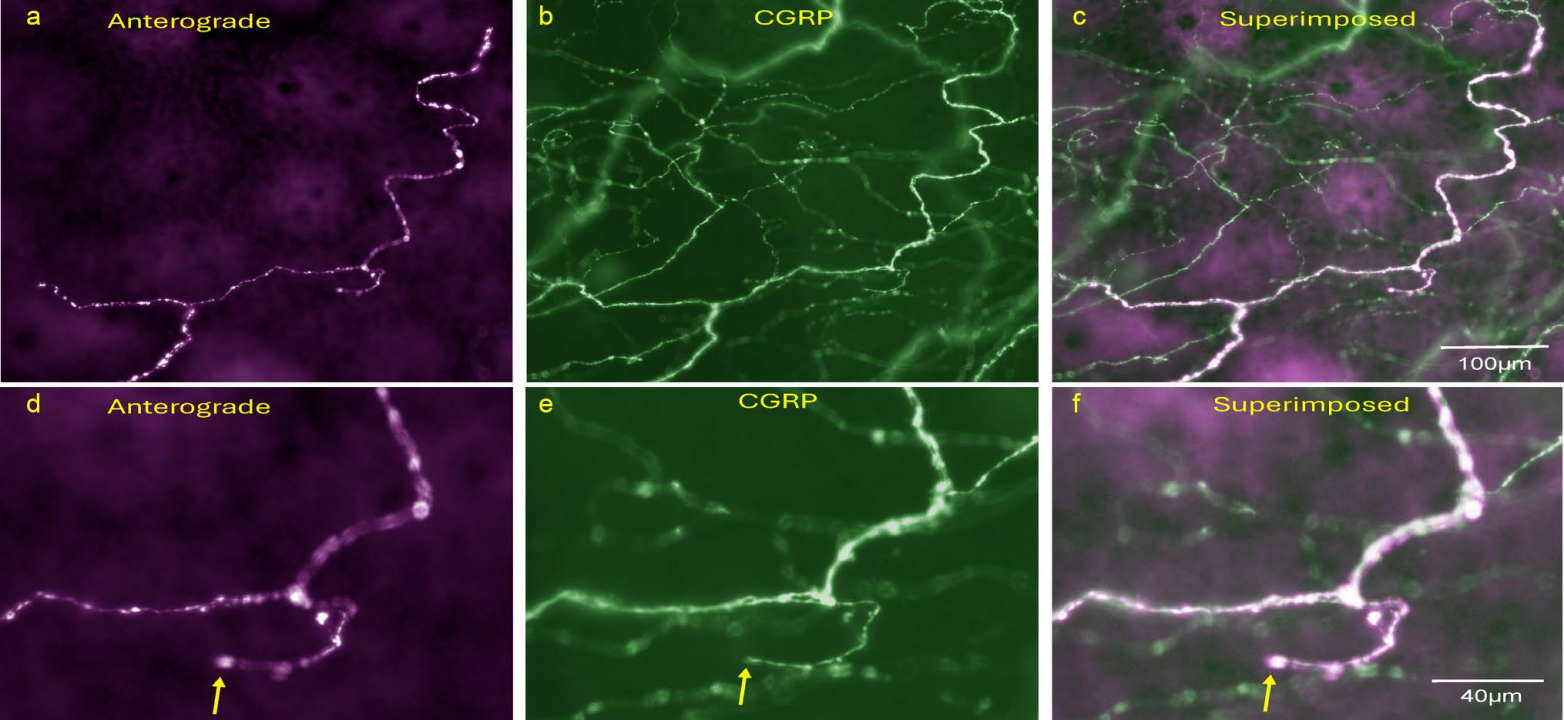
Simple type spinal afferent in the sub/urothelium.** a** A single varicose axon bifurcates to form three simple terminal endings. Axon diameter remains consistent after branching, with small varicosities at each terminus. **b** CGRP immunoreactivity of the same field shows multiple labelled axons in the sub/urothelium. **c** Overlay of **a** and **b**. **d** Enlarged view of the afferent in **a**; the arrow indicates a simple varicose terminal. **e** CGRP immunoreactivity corresponding to **d**. **f** Overlay of **d** and **e**; arrows confirm the ending is CGRP-positive (peptidergic)

**Fig. 3:**
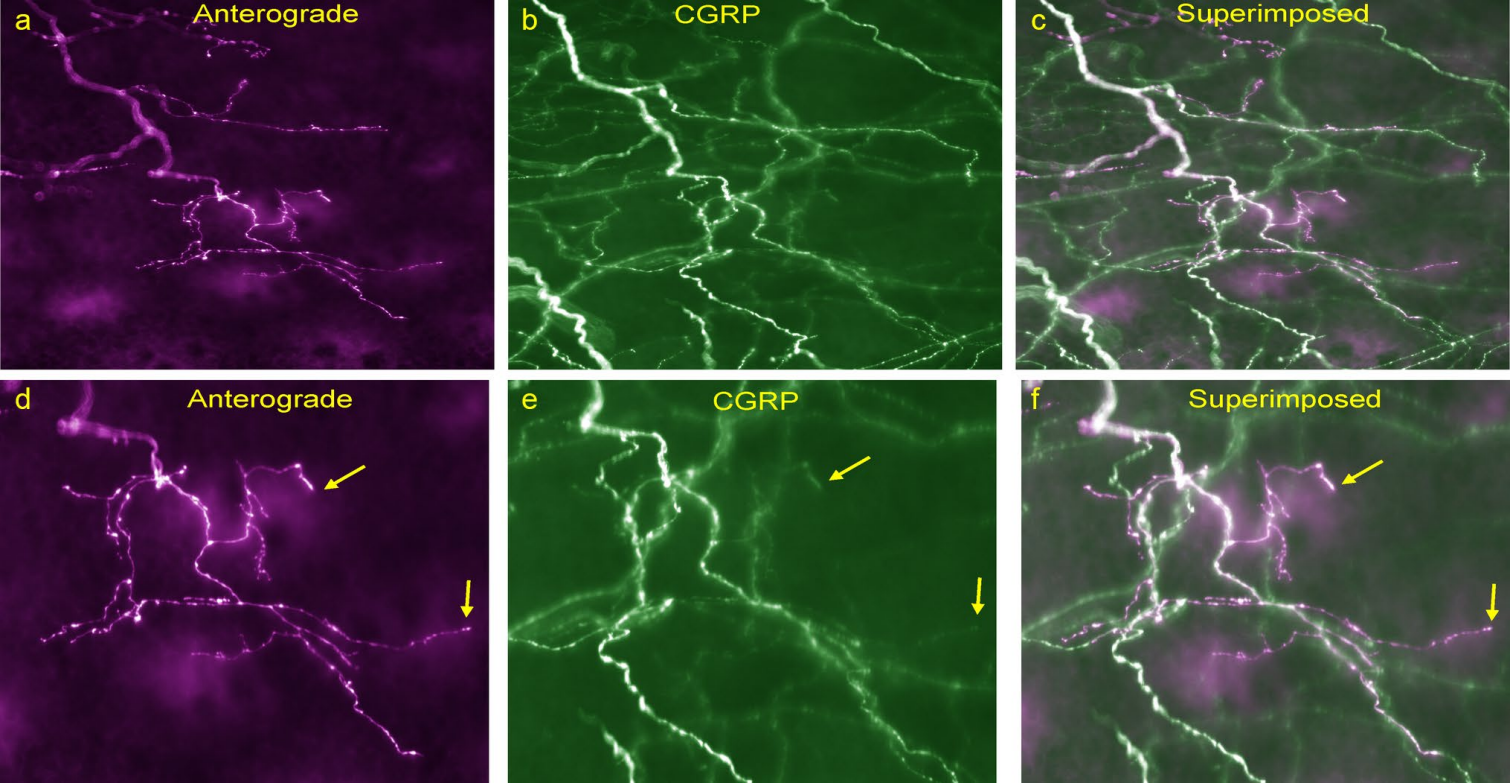
Peptidergic complex-type spinal afferent ending in the sub/urothelial layer.** a** The complex-type ending. **b** CGRP immunoreactivity of the field in **a**. **c** Overlay of **a** and **b**. **d** Enlarged view from **a**; arrows indicate two terminal endings. **e** CGRP immunoreactivity of the field in **d**; arrows mark the corresponding CGRP-positive endings. **f** Overlay of **d** and **e** confirming colocalization

Anterograde labelling of bilateral T12–L3 DRGs revealed multiple morphological classes of single-axon nerve endings in the urinary bladder. We identified 24 distinct endings from 8 mice, categorizing them into four types: two within the detrusor muscle and two in the sub/urothelium. Most endings (19/24) were in the sub/urothelium layer (present in 7/8 mice), with the simple type being the most prevalent (12/24). These simple endings comprised single axons that ramified near urothelial cells with minimal branching and no preferential alignment to the bladder wall or specific association with urothelial cells. Varicosities were present along the axons, including at their terminals, which lacked any specialized morphological structures (Figs. 1, 2, and 3). All simple-type endings were immunoreactive for CGRP (Figs. 1, 2, and 3).

**Fig. 4:**
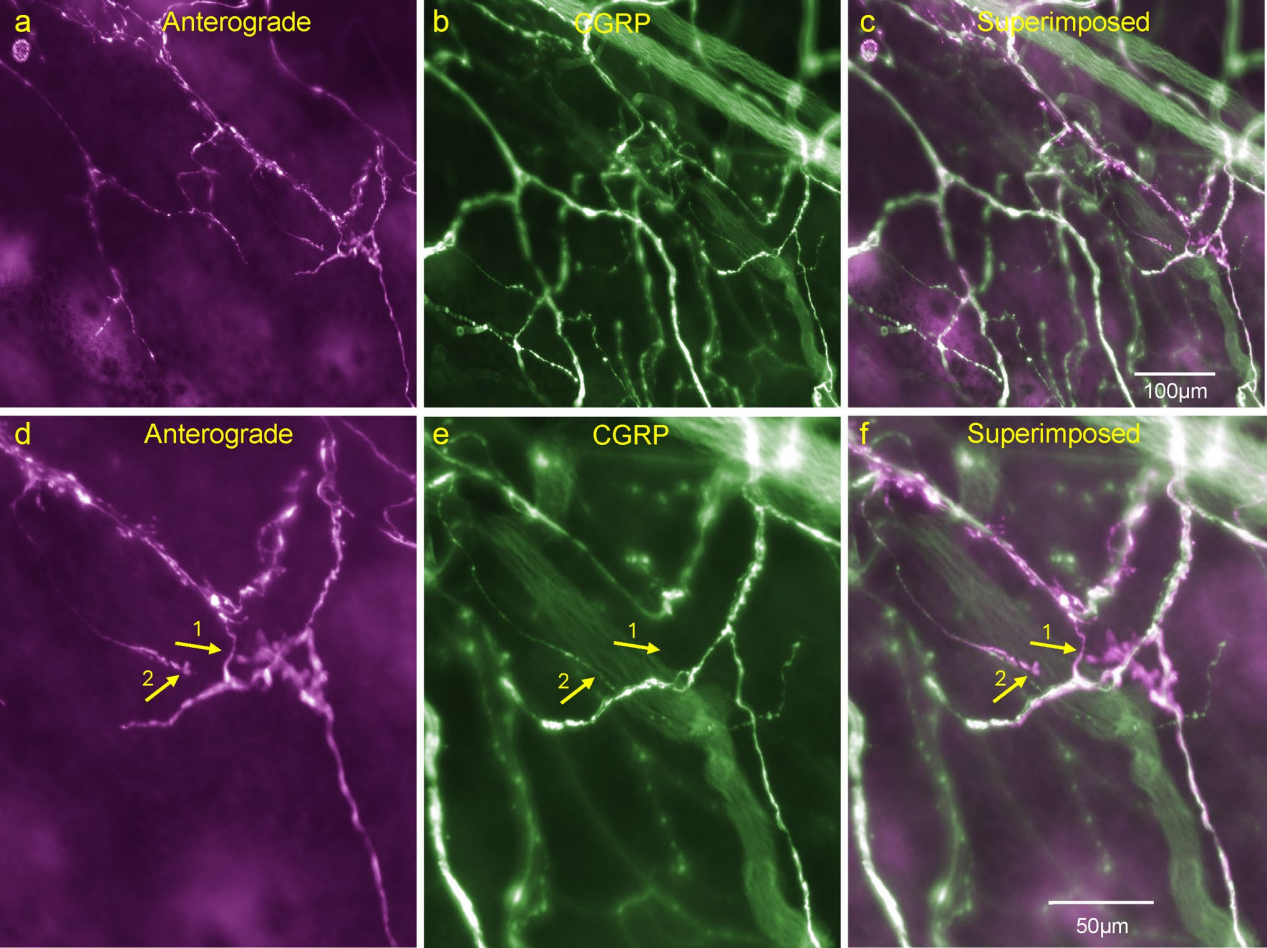
Non-peptidergic “complex-type” spinal afferent ending in the sub/urothelium layer.** a** The complex-type ending. **b** CGRP immunoreactivity of the field in **a**, showing an absence of labelling. **c** Overlay of **a** and **b**. **d** Enlarged view from **a**; arrow 1 indicates an axon and arrow 2 a nerve ending. **e** Corresponding CGRP immunoreactivity of **d**, confirming the lack of labelling at both locations (arrows 1 and 2). **f** Overlay of **d** and **e**

Within the sub/urothelium, we classified 7 of the 19 endings as complex-type (Figs. 3 and 4; Table 2). These comprised multiple bifurcating axons with short, varicose side branches (Fig. 4). Their axon diameters and varicosity sizes did not differ from other ending classes (Table 2). All were CGRP-immunoreactive (Fig. 3), except for one non-peptidergic example (Fig. 4).

**Table 2:** Morphological types and layers

					Mean ± SD		
Location	Type	Count (%)	Animals (%)/sex	CGRP + (%)	Axon diam	Varic. length	Varic. width
Sub/urothelium	Simple	12 (50)	4 (50)/female	12 (100)	1.32 ± 0.35	2.14 ± 0.49	1.42 ± 0.29
Sub/urothelium	Complex	7 (29)	3 (37)/female	6 (86)	1.51 ± 0.87	2.51 ± 0.79	1.64 ± 0.77
Detrusor	Simple	4 (17)	3 (37)/female	4 (100)	1.47 ± 0.23	2.05 ± 0.22	1.37 ± 0.17
Detrusor	Complex	1 (4)	1 (12)/female	1 (100)	3.06	2.58	1.56

**Fig. 5:**
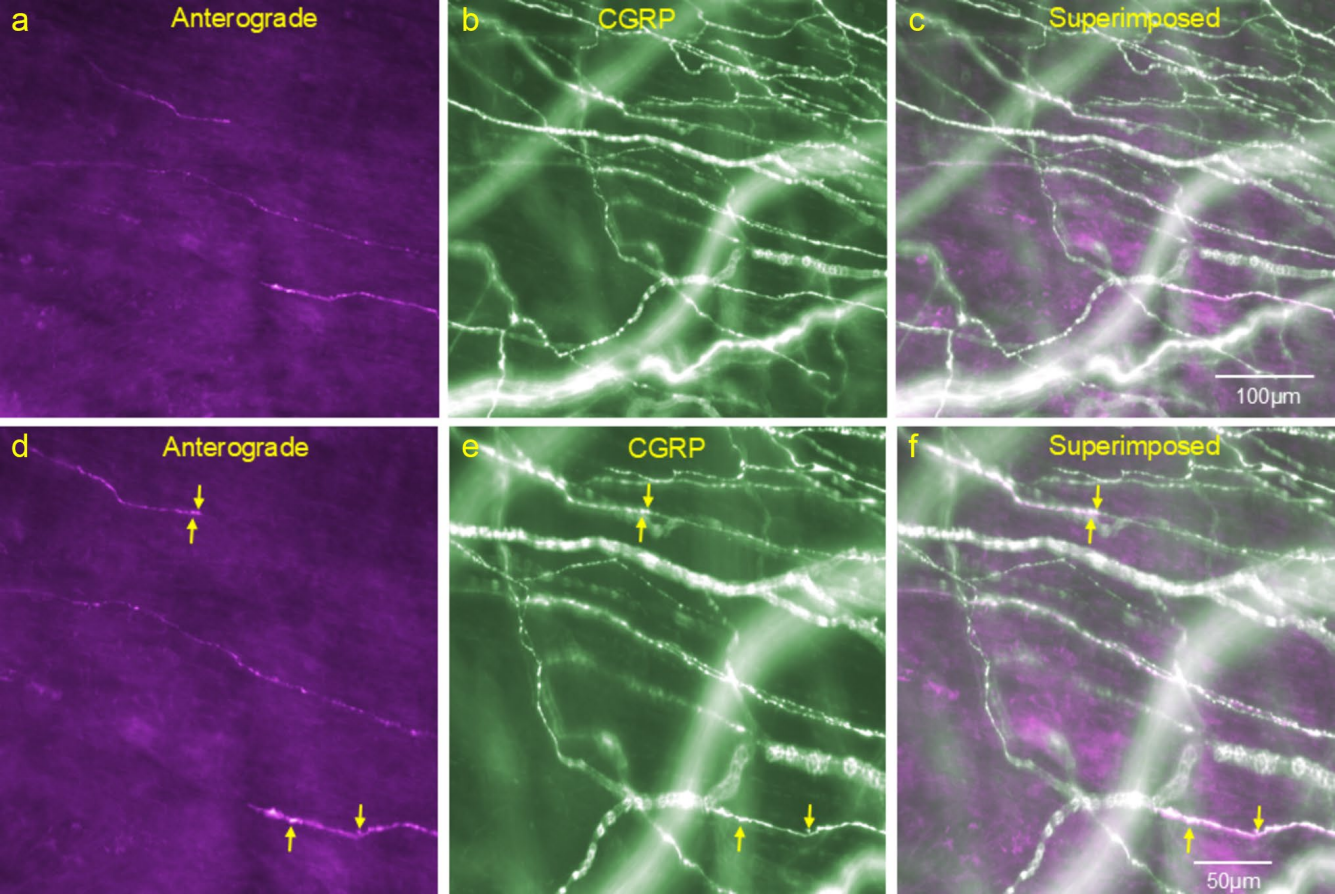
Simple peptidergictype ending in the detrusor muscle.** a** Anterogradely labelled axons and endings. **b** CGRP immunoreactivity of the field in **a**. **c** Overlay of **a** and **b**. **d** Enlarged view from **a**. **e** CGRP immunoreactivity of the field in **d**. **f** Overlay of **d** and **e** confirming colocalization

In the detrusor muscle, we identified 5 endings (4 mice). The simple type was again most common (4/5), with one complex-type ending (Table 2; Fig. 5). All detrusor endings were immunoreactive for CGRP. A notable finding was an absence of branching-type endings in the detrusor, contrasting with our earlier lumbosacral DRG study (see Fig. 6 in Spencer et al. (2018)). Overall, varicosity dimensions and the diameters of parent axons showed no significant differences across morphological types or between layers (Table 2).

## Discussion

**Fig. 6:**
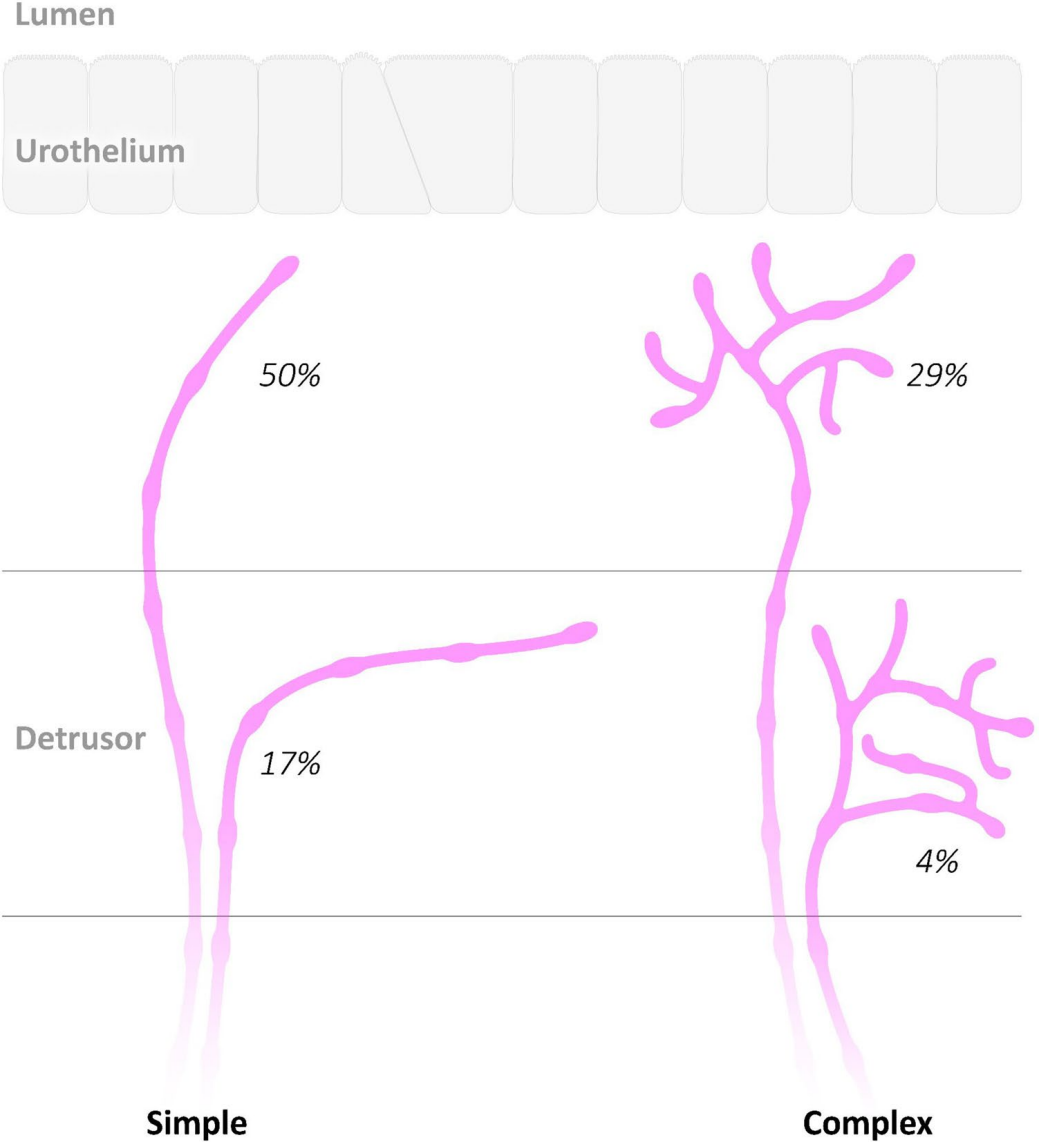
Graphical representation of the different types of spinal afferent endings in the urinary bladder that arise from thoracolumbar DRG. Both simple and complex types were identified in both the sub/urothelium and in the detrusor muscle. But the distribution was significantly different. The majority of TL afferents (79%) terminated within the sub/urothelium. Only a relatively small proportion (21%) innervated the detrusor

This study has identified the morphological types of spinal afferent nerve endings in the urinary bladder that originate from thoracolumbar DRG (T12–L3). We characterized four types, mirroring those from lumbosacral DRG (Spencer et al. 2018): simple and complex endings in both the sub/urothelium and detrusor muscle (Fig. 6). However, their distribution was markedly different. In contrast to the predominant detrusor innervation (81%) from lumbosacral levels, the majority (79%) of thoracolumbar afferents terminated within the sub/urothelium.

The population-specific bias was also reflected in labelling efficiency: thoracolumbar labelling in this study yielded an average of 2.4 sub/urothelium and 0.6 detrusor afferents per animal, whereas lumbosacral labelling produced 1.0 and 4.2 afferents per animal for the same targets (Spencer et al. 2018), respectively. This suggests that the proportional differences in target innervation reflect a change in the frequency of afferent labelling for both targets, not just a shift in preference for one. Interestingly, this pattern may be reversed in the rat, where retrograde tracing showed a higher proportion of sub/urothelium afferents in lumbosacral pathways, most of which lacked neuropeptides like CGRP and substance P (Clodfelder-Miller et al. 2023), suggesting potential species differences in the organization of spinal afferent pathways. The diameter of spinal afferent axons within the bladder was similar to measurements of sensory or motor axons identified using electron microscopy in the rat urinary bladder (Gabella 1999). It is important to note that our measurements are approximate morphometric estimates, not absolute structural dimensions.

We were surprised to note an absence of branching-type endings in the detrusor muscle in the current study. This is quite different from our earlier study where branching-type endings were prevalent in the detrusor muscle occupying 12% of the total population of spinal afferent endings identified, after labelling from lumbosacral DRG (Spencer et al. 2018). It is possible that this type of nerve ending exists in the detrusor muscle from sensory neurons arising from thoracolumbar DRG, but the relatively lower numbers of animals injected in this study precluded their identification. It is also possible there are differences in the efficiency of tracer uptake between thoracolumbar and lumbosacral DRG which may represent a technical limitation when comparing populations of spinal afferents across spinal levels.

The proportion of peptidergic (CGRP +) thoracolumbar afferents (96%) was higher than in our previous study of lumbosacral afferents (87%). This aligns with transcriptomic data suggesting more classes of lumbosacral bladder afferents have low or no Calca expression (Meerschaert et al. 2020) and with findings in colonic afferents (Christianson & Davis 2010). As before, all “simple” type endings were CGRP + (Spencer et al. 2018). The non-peptidergic population, found exclusively among “complex” and “branching” types (this study and (Spencer et al. 2018), may include recently described TrkB-expressing, putatively mechanosensitive bladder afferents that also lack CGRP (Tran et al. 2025).

Xu and Gebhart made electrophysiological recordings from bladder sensory fibers in the lumbar splanchnic and pelvic afferent pathways in mice (Xu & Gebhart 2008). They identified 3 functional types of afferents in the lumbar splanchnic pathway and 4 types in the pelvic afferent pathway (Xu & Gebhart 2008). In the thoracolumbar splanchnic pathway, mechanosensitive afferents were topographically clustered to the base of the bladder, while mechanosensitive pelvic afferents were distributed throughout the bladder. Guinea pig bladder mucosal (sub/urothelium) afferents have also been characterized in detail (Zagorodnyuk et al. 2006; Zagorodnyuk et al. 2007). They respond to fine mechanical compression of the mucosa but not to bladder stretch. It is likely that some of the morphological types of endings identified arising from T12-L3 DRG in our study give rise to mucosal afferents described previously (Zagorodnyuk et al. 2009; Zagorodnyuk et al. 2006, 2007). Indeed, two classes of bladder afferents have been studied using electrophysiological recordings that had their endings in the vicinity of the sub/urothelium (Zagorodnyuk et al. 2009). These were functionally classified as stretch-sensitive muscular-mucosal mechanoreceptors and stretch-insensitive, mucosal high-responding afferents (Zagorodnyuk et al. 2009). It is tempting to speculate that the two distinct morphological types of spinal afferent endings we identified in the sub/urothelium in the current study may reflect the two distinct functional classes of mucosal bladder afferents identified functionally that could be separated based on mechanical sensitivities or to distinguish between mucosal and muscular-mucosal afferent classes (Zagorodnyuk et al. 2010; Zagorodnyuk et al. 2009).

The specific mechanisms by which sub/urothelium stimuli activate the identified sensory endings remain unclear. While urothelial cells are known transducers of various stimuli (Birder 2010; Birder et al. 2010; Grundy et al. 2018), communication with afferent endings is unlikely to involve conventional calcium-dependent synaptic transmission, as mechanical responses persist in calcium-free conditions (Zagorodnyuk et al. 2009). Whether direct physical contacts exist between urothelial cells and spinal afferent endings requires further investigation. This question is analogous to the original hypothesis in the gastrointestinal tract, where enterochromaffin (EC) cells were initially proposed to form fast synaptic connections with afferents (Bellono et al. 2017; Kaelberer et al. 2018), but recent studies have found no evidence of such direct morphological contacts for either spinal (Dodds et al. 2022) or vagal afferent endings (Spencer et al. 2024).

In conclusion, while spinal afferent neurons from thoracolumbar (T12–L3) and lumbosacral DRG form morphologically similar ending types in the urinary bladder, they exhibit a profound and distinct innervation bias. Most thoracolumbar afferents are peptidergic and target the sub/urothelium, whereas lumbosacral afferents primarily innervate the detrusor muscle (Spencer et al. 2018). This anatomical segregation, coupled with the absence of branching-type endings in the thoracolumbar-detrusor pathway, strongly suggests that these two spinal afferent populations are functionally specialized to detect and relay different categories of sensory information from the bladder (Fig. 6).

## Data Availability

All data is freely available upon request from the corresponding author.

## References

[CR1] Bellono NW, Bayrer JR, Leitch DB, Castro J, Zhang C, O’Donnell TA, O’Donnell TA, Brierley SM, Ingraham HA, Julius D (2017) Enterochromaffin cells are gut chemosensors that couple to sensory neural pathways. Cell 170(1):185-198 e116. 10.1016/j.cell.2017.05.034 28648659 10.1016/j.cell.2017.05.034 PMC5839326

[CR2] Birder LA (2010) Urothelial signaling. Auton Neurosci 153(1–2):33–40. 10.1016/j.autneu.2009.07.005 19666243 10.1016/j.autneu.2009.07.005 PMC2818048

[CR3] Birder LA, Wolf-Johnston AS, Chib MK, Buffington CA, Roppolo JR, Hanna-Mitchell AT (2010) Beyond neurons: involvement of urothelial and glial cells in bladder function. Neurourol Urodyn 29(1):88–96. 10.1002/nau.20747 20025015 10.1002/nau.20747 PMC2910110

[CR4] Christianson JA & Davis BM (2010) The role of visceral afferents in disease. In L. Kruger & A. R. Light (Eds.), Translational Pain Research: From Mouse to Man. Boca Raton, FL.

[CR5] Clodfelder-Miller B, DeBerry JJ, Ness TJ (2023) Urothelial bladder afferents selectively project to L6/S1 levels and are more peptidergic than those projecting to the T13/L1 levels in female rats. Heliyon 9(8). 10.1016/j.heliyon.2023.e18495 37534006 10.1016/j.heliyon.2023.e18495 PMC10392082

[CR6] Dodds KN, Travis L, Kyloh MA, Jones LA, Keating DJ, Spencer NJ (2022) The gut-brain axis: spatial relationship between spinal afferent nerves and 5-HT-containing enterochromaffin cells in mucosa of mouse colon. Am J Physiol Gastrointest Liver Physiol 322(5):G523–G533. 10.1152/ajpgi.00019.2022 35293258 10.1152/ajpgi.00019.2022

[CR7] Gabella G (1999) Structure of the intramural nerves of the rat bladder. J Neurocytol 28(8):615–637. 10.1023/a:1007084130642 10851342 10.1023/a:1007084130642

[CR8] Grundy L, Chess-Williams R, Brierley SM, Mills K, Moore KH, Mansfield K, Rose’Meyer R, Sellers D, Grundy D (2018) NKA enhances bladder afferent mechanosensitivity via urothelial and detrusor activation. Am J Physiol Renal Physiol. 10.1152/ajprenal.00106.2018 29897284 10.1152/ajprenal.00106.2018 PMC6230738

[CR9] Grundy L, Harrington AM, Caldwell A, Castro J, Staikopoulos V, Zagorodnyuk VP, Brookes SJH, Spencer NJ, Brierley SM (2019) Translating peripheral bladder afferent mechanosensitivity to neuronal activation within the lumbosacral spinal cord of mice. Pain 160(4):793–804. 10.1097/j.pain.0000000000001453 30531372 10.1097/j.pain.0000000000001453

[CR10] Janig W (1986) Spinal cord integration of visceral sensory systems and sympathetic nervous system reflexes. Prog Brain Res 67:255–277. 10.1016/s0079-6123(08)62767-3 3823476 10.1016/s0079-6123(08)62767-3

[CR11] Janig W, Morrison JF (1986) Functional properties of spinal visceral afferents supplying abdominal and pelvic organs, with special emphasis on visceral nociception. Prog Brain Res 67:87–114. 10.1016/s0079-6123(08)62758-2 3823484 10.1016/s0079-6123(08)62758-2

[CR12] Kaelberer MM, Buchanan KL, Klein ME, Barth BB, Montoya MM, Shen X, Bohorquez DV (2018) A gut-brain neural circuit for nutrient sensory transduction. Science. 10.1126/science.aat5236 30237325 10.1126/science.aat5236 PMC6417812

[CR13] Kyloh M, Spencer NJ (2014) A novel anterograde neuronal tracing technique to selectively label spinal afferent nerve endings that encode noxious and innocuous stimuli in visceral organs. Neurogastroenterol Motil 26(3):440–444. 10.1111/nmo.12266 24460783 10.1111/nmo.12266

[CR14] Meerschaert KA, Adelman PC, Friedman RL, Albers KM, Koerber HR, Davis BM (2020) Unique molecular characteristics of visceral afferents arising from different levels of the neuraxis: location of afferent somata predicts function and stimulus detection modalities. J Neurosci 40(38):7216–7228. 10.1523/JNEUROSCI.1426-20.2020 32817244 10.1523/JNEUROSCI.1426-20.2020 PMC7534907

[CR15] Spencer NJ, Greenheigh S, Kyloh M, Hibberd TJ, Sharma H, Grundy L, Brierley SM, Harrington AM, Beckett EA, Brookes SJ, Zagorodnyuk VP (2018) Identifying unique subtypes of spinal afferent nerve endings within the urinary bladder of mice. J Comp Neurol 526(4):707–720. 10.1002/cne.24362 29178500 10.1002/cne.24362

[CR16] Spencer NJ, Kyloh M, Duffield M (2014) Identification of different types of spinal afferent nerve endings that encode noxious and innocuous stimuli in the large intestine using a novel anterograde tracing technique. PLoS ONE 9(11). 10.1371/journal.pone.0112466 25383884 10.1371/journal.pone.0112466 PMC4226564

[CR17] Spencer NJ, Kyloh MA, Travis L, Hibberd TJ (2024) Identification of vagal afferent nerve endings in the mouse colon and their spatial relationship with enterochromaffin cells. Cell Tissue Res 396(3):313–327. 10.1007/s00441-024-03879-6 38383905 10.1007/s00441-024-03879-6 PMC11144134

[CR18] Tran EL, Stuedemann SA, Ridlon M, Link OD, Keil Stietz KP, Crawford LK (2025) Genetic tools that target mechanoreceptors produce reliable labeling of bladder afferents and altered mechanosensation. Am J Physiol Renal Physiol 328(3):F360–F374. 10.1152/ajprenal.00151.2024 39611874 10.1152/ajprenal.00151.2024 PMC12192779

[CR19] Xu L, Gebhart GF (2008) Characterization of mouse lumbar splanchnic and pelvic nerve urinary bladder mechanosensory afferents. J Neurophysiol 99(1):244–253 1152/jn.01049.2007 18003875 10.1152/jn.01049.2007 PMC2659401

[CR20] Zagorodnyuk VP, Brookes SJ, Spencer NJ (2010) Structure-function relationship of sensory endings in the gut and bladder. Auton Neurosci 153(1–2):3–11. 10.1016/j.autneu.2009.07.018 19682956 10.1016/j.autneu.2009.07.018

[CR21] Zagorodnyuk VP, Brookes SJ, Spencer NJ, Gregory S (2009) Mechanotransduction and chemosensitivity of two major classes of bladder afferents with endings in the vicinity to the urothelium. J Physiol 587(Pt 14):3523–3538 19470774 10.1113/jphysiol.2009.172577 PMC2742279

[CR22] Zagorodnyuk VP, Chen BN, Brookes SJ (2001) Intraganglionic laminar endings are mechano-transduction sites of vagal tension receptors in the guinea-pig stomach. J Physiol 534(Pt 1):255–268 11433006 10.1111/j.1469-7793.2001.00255.x PMC2278677

[CR23] Zagorodnyuk VP, Costa M, Brookes SJ (2006) Major classes of sensory neurons to the urinary bladder. Auton Neurosci 126–127:390–397 (doi:S1566-0702(06)00054-3 [pii]) 16581309 10.1016/j.autneu.2006.02.007

[CR24] Zagorodnyuk VP, Gibbins IL, Costa M, Brookes SJ, Gregory SJ (2007) Properties of the major classes of mechanoreceptors in the guinea pig bladder. J Physiol 585(Pt 1):147–163. 10.1113/jphysiol.2007.140244 17916614 10.1113/jphysiol.2007.140244 PMC2375472

